# ATXN3 functions as a tumor suppressor through potentiating galectin-9-mediated apoptosis in human colon adenocarcinoma

**DOI:** 10.1016/j.jbc.2024.107415

**Published:** 2024-05-28

**Authors:** Yang Cheng, Shengnan Wang, Qiong Gao, Deyu Fang

**Affiliations:** 1 Department of Pathology, Northwestern University Feinberg School of Medicine, Chicago, Illinois, USA; 2 Robert H. Lurie Comprehensive Cancer Center, Northwestern University Feinberg School of Medicine, Chicago, Illinois, USA; 3 Center for Human Immunology, Northwestern University Feinberg School of Medicine, Chicago, Illinois, USA

**Keywords:** ATXN3, galectin-9, colon cancer, deubiquitinase, cancer apoptosis

## Abstract

While deubiquitinase ATXN3 has been implicated as a potential oncogene in various types of human cancers, its role in colon adenocarcinoma remains understudied. Surprisingly, our findings demonstrate that ATXN3 exerts an antitumor effect in human colon cancers through potentiating Galectin-9-induced apoptosis. CRISPR-mediated ATXN3 deletion unexpectedly intensified colon cancer growth both *in vitro* and in xenograft colon cancers. At the molecular level, we identified ATXN3 as a *bona fide* deubiquitinase specifically targeting Galectin-9, as ATXN3 interacted with and inhibited Galectin-9 ubiquitination. Consequently, targeted ATXN3 ablation resulted in reduced Galectin-9 protein expression, thereby diminishing Galectin-9-induced colon cancer apoptosis and cell growth arrest. The ectopic expression of Galectin-9 fully reversed the growth of ATXN3-null colon cancer in mice. Furthermore, immunohistochemistry staining revealed a significant reduction in both ATXN3 and Galectin-9 protein expression, along with a positive correlation between them in human colon cancer. Our study identifies the first Galectin-9-specific deubiquitinase and unveils a tumor-suppressive role of ATXN3 in human colon cancer.

Colon adenocarcinoma (COAD) stands as the predominant pathological variant within colon cancer, ranking as the second most prevalent cause of global cancer-related fatalities. In the year 2020, an estimated 1.93 million new cases of colorectal cancer were diagnosed, leading to 0.94 million deaths attributed to colorectal cancer worldwide. This accounts for 10% of the total global cancer incidence (comprising 19.29 million new cases) and 9.4% of all cancer-related deaths (totaling 9.96 million deaths). Notably, both the incidence and mortality rates of COAD have exhibited a consistent upward trajectory, primarily linked to the unfavorable prognosis associated with advanced COAD cases. Numerous ubiquitin-specific peptidases (USPs), or deubiquitinases, have been identified to play pivotal oncogenic roles in colon cancer. The deubiquitinase USP25, for instance, supports colonic inflammation and bacterial infection, promoting colorectal cancer through Wnt and SOCS3-pSTAT3 signaling (1). Several other deubiquitinases, including USP10 (2), USP22 (3, 4), USP28 (5), USP7, and CYLD (6), have been demonstrated to foster the development of colon cancer by activating oncogenic Myc. Furthermore, our research and others have revealed the critical role of USP22 in promoting colon cancer cell growth and inhibiting apoptosis through p53 suppression and the stabilization of cyclins B1 and D1 (3, 7). Similarly, USP17 exhibits high expression in colon cancer biopsies, is cell cycle-regulated, and is essential for G1-S progression (8). Moreover, deubiquitinases from the OTUD family, such as OTUD1 (9, 10), OTUD3 (11), and OTUD4 (12), have been implicated in colon inflammation and cancer. In contrast, USP9x has been found to stabilize the E3 ubiquitin ligase Fbw7, inhibiting Myc as a tumor suppressor (13).

ATXN3, also known as Ataxin-3, ATX3, AT3, or MJD, belongs to the MJD family, which is one of the five primary cysteine deubiquitinating enzyme families. The physiological functions of ATXN3 are largely unexplored. Mice with targeted deletion of the ATXN3 gene throughout their systems are viable, fertile, and do not exhibit a reduced lifespan (14). Numerous studies have highlighted the cell-autonomous role of ATXN3 in tumorigenesis. Although direct substrates were not initially identified, ATXN3 has been implicated in restraining PTEN transcription in lung cancer cells (15). Furthermore, in breast cancer, ATXN3 promotes metastasis by deubiquitinating KLF4, a transcription factor frequently overexpressed in various human cancers and closely associated with tumorigenesis and tumor progression (16). Recently, both our research and Wu *et al.* have unveiled ATXN3's involvement in driving prostate cancer progression by stabilizing YAP (17, 18). Additionally, our recent CRISPR screening identified ATXN3 as a positive regulator of the immune checkpoint receptor PD-L1, facilitating evasion of tumor immunosurveillance (19). However, the role of ATXN3 in the development and progression of colon cancer remains largely unexplored.

Contrary to our expectations, targeted ablation of the ATXN3 gene resulted in a striking increase in colon cancer growth. Immunohistochemical staining indicated a decrease in ATXN3 levels in human colon adenocarcinoma compared to those in healthy colon tissues, implying a tumor-suppressive role for ATXN3. Intriguingly, ATXN3 acts as an endogenous deubiquitinase of Galectin-9, thereby targeting ATXN3 deletion resulting in the reduced Galectin-9 expression and apoptosis of colon cancer cells. Our study unveils a previously unknown tumor-suppressive function of ATXN3 in the progression of colon cancer.

## Results

### ATXN3 promotes Galectin-9 expression in human colon cancer cells

**Figure 1:**
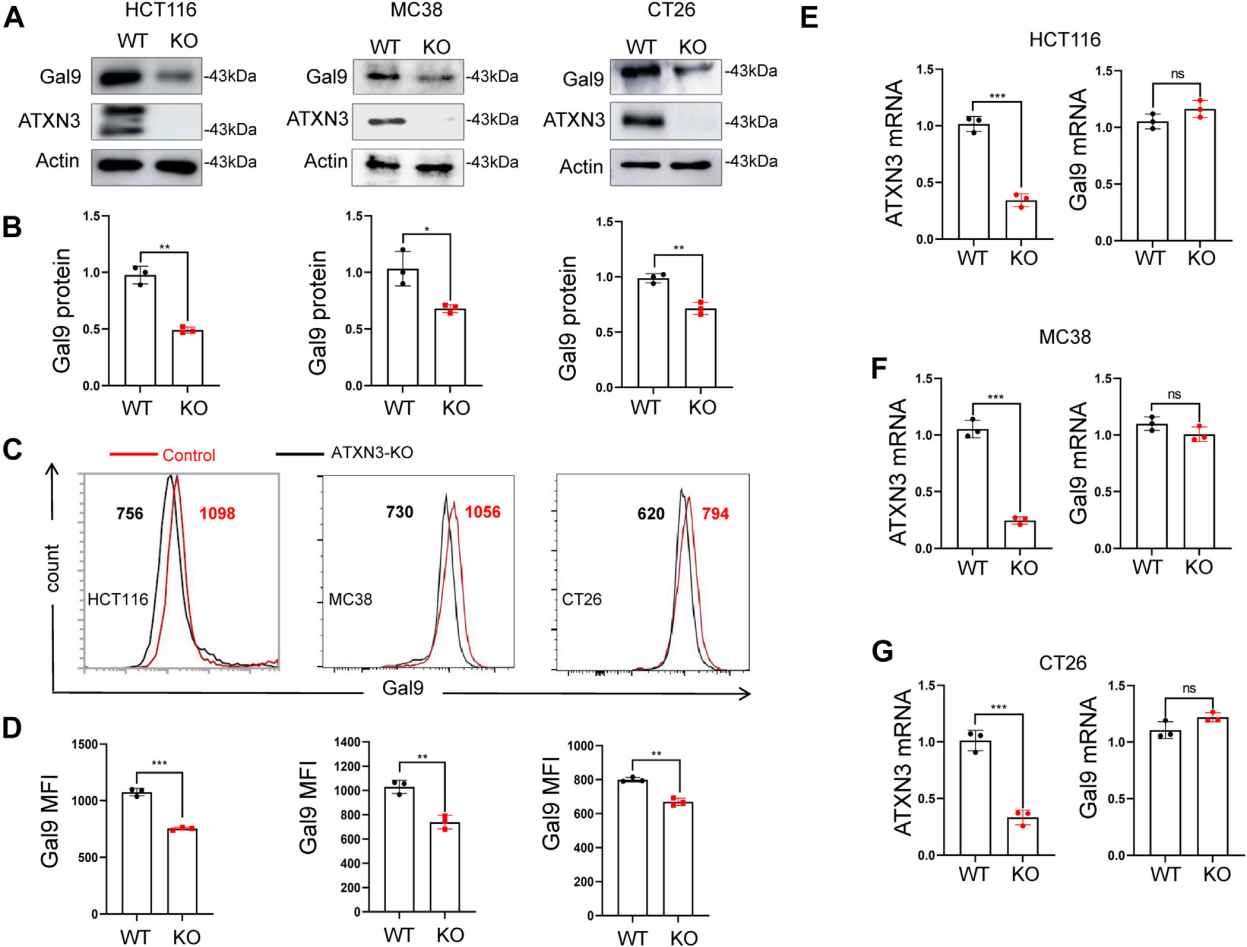
**ATXN3 promotes Galectin-9 expression in human colon cancer cells.***A*, ATXN3 targeted deletion in different colon cancer cells were generated. The protein levels of ATXN3(*top panel*), Galectin-9 (*middle panel*), and loading control β-actin were determined by western blotting. *B*, the levels of Galectin-9 in (*A*) and repeated experiments were quantified using Image J software. *C* and *D*, representative flow cytometry plots and quantification by MFI of Galectin-9 in HCT116, MC38, and CT26 cells. *E*, ATXN3 and Galectin-9 mRNA levels were analyzed by reverse transcription quantitative PCR (RT -qPCR) in HCT116 cells. *F*, ATXN3 and Galectin-9 mRNA levels were analyzed by reverse transcription quantitative PCR (RT -qPCR) in MC38 cells. *G*, ATXN3 and Galectin-9 mRNA levels were analyzed by reverse transcription quantitative PCR (RT -qPCR) in CT26 cells. *B*, *D*, *E*, *F* and *G*: 2-tailed unpaired *t* test; ∗*p* < 0.05, ∗∗*p* < 0.01, ∗∗∗*p* < 0.001.

In our recent CRISPR screening, ATXN3 emerged as a positive regulator of PD-L1 (19). To investigate the potential role of ATXN3 in governing the tumoral expression of immune modulatory molecules, we created ATXN3 knockout colon cancer cells, including HCT116, CT26, and MC38. Western blotting analysis unequivocally confirmed the thorough deletion of the ATXN3 gene. Intriguingly, the targeted removal of the ATXN3 gene led to a noteworthy decrease in the protein expression of Galectin-9 (Fig. 1, *A* and *B*), a carbohydrate-binding protein known for modulating immune response, inhibiting tumor cell growth, and inducing apoptosis (20). Subsequent flow cytometry analysis corroborated a modest yet statistically significant reduction in the cell surface expression of Galectin-9 in colon cancer cells (Fig. 1, *C* and *D*). Crucially, real-time RT-PCR analysis failed to detect any decrease in Galectin-9 mRNA expression levels (Fig. 1, *E*–*G*), underscoring that ATXN3 regulates Galectin-9 protein expression at a post-transcriptional level.

### Galectin-9 is an endogenous substrate for the ATXN3 deubiquitinase

**Figure 2:**
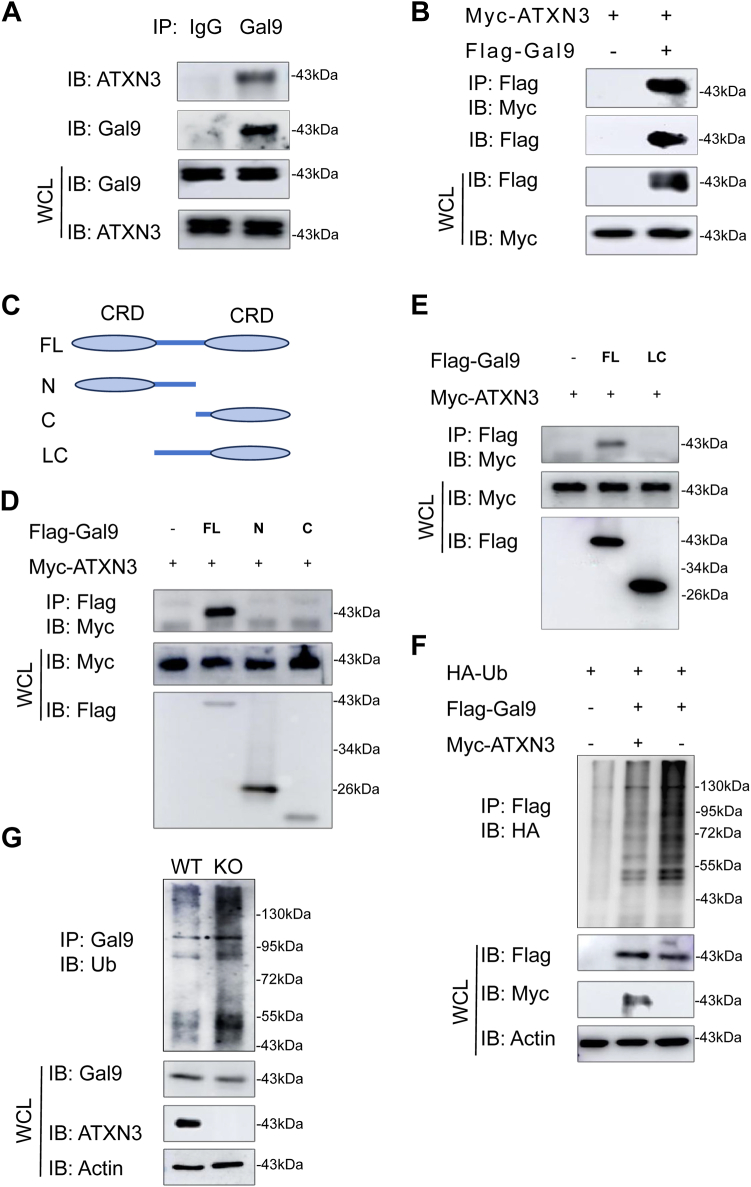
**Galectin-9 is an endogenous substrate for the ATXN3 deubiquitinase.***A*, interaction of endogenous ATXN3 and Galectin-9 in HCT116 cells. *B*, ATXN3 interacts with Galectin-9 in transiently transfected in HEK293T cells. *C*, schematic representation of Galectin-9 and its mutants, showing that Galectin-9 protein contains an N-terminal carbohydrate recognition threshold (CRD), a C-terminal CRD, and a peptide junction domain. *D* and *E*, the interactions of Galectin-9 and its truncated mutants with ATXN3 protein were tested. ATXN3 plasmids were cotransfected with Galectin-9 or each of its mutants and their interactions were analyzed as in (*B*). *F*, HA-ubiquitin and Flag-Galectin-9 expression plasmids were cotransfected with Myc-ATXN3 into HEK293T cells. Galectin-9 ubiquitination was determined by immunoprecipitation of Galectin-9 and immunoblotting with HA antibody. *G*, the ubiquitination of endogenous Galectin-9 was analyzed by immunoprecipitation with an anti-Galectin-9 antibody and western blotting with an anti-ubiquitin antibody in the WT and ATXN3 knockout HCT116 cell line. WCL, whole-cell lysate.

Our findings that ATXN3 ablation led to a decrease in Galectin-9 protein expression without altering its mRNA levels suggest that ATXN3 regulates Galectin-9 protein expression at the post-transcriptional level. Substantiating this, co-immunoprecipitation (co-IP) and Western blotting revealed an interaction between ATXN3 and Galectin-9 in transiently transfected HEK293 cells (Fig. 2*B*). Consistently, ATXN3 protein was identified through Western blotting in anti-Galectin-9 immunoprecipitants but not in normal rabbit IgG controls from HCT116 colon cancer cells (Fig. 2*A*). Galectin-9 has two carbohydrate recognition domains with a linker region in between (Fig. 2*C*). Interestingly, neither the N-terminal nor the C-terminal galectin domain displayed the ability to interact with ATXN3, implying that either the linker region or the entire Galectin-9 protein mediates its interaction with ATXN3 (Fig. 2*D*). We then further generated a truncated mutant with the whole linker region and the C-terminal carbohydrate recognition domains (Fig. 2*C*), but its interaction with ATXN3 was still not detected (Fig. 2*E*), indicating that the entire Galectin-9 protein is required to mediate its interaction with ATXN3.

As a deubiquitinase typically inhibits ubiquitin-conjugation to its interacting protein, strong high molecular weight bands were observed in Galectin-9 immunoprecipitants with anti-HA-Ub when both were transfected into HEK293 cells. Co-expression of ATXN3 significantly inhibited Galectin-9 ubiquitination (Fig. 2*F*). Conversely, ATXN3 ablation resulted in a significant increase in Galectin-9 protein ubiquitination (Fig. 2*G*). Thus, these results indicate that ATXN3 functions as a Galectin-9 deubiquitinase in colon cancer cells.

### ATXN3 protects Galectin-9 from proteasomal degradation

**Figure 3:**
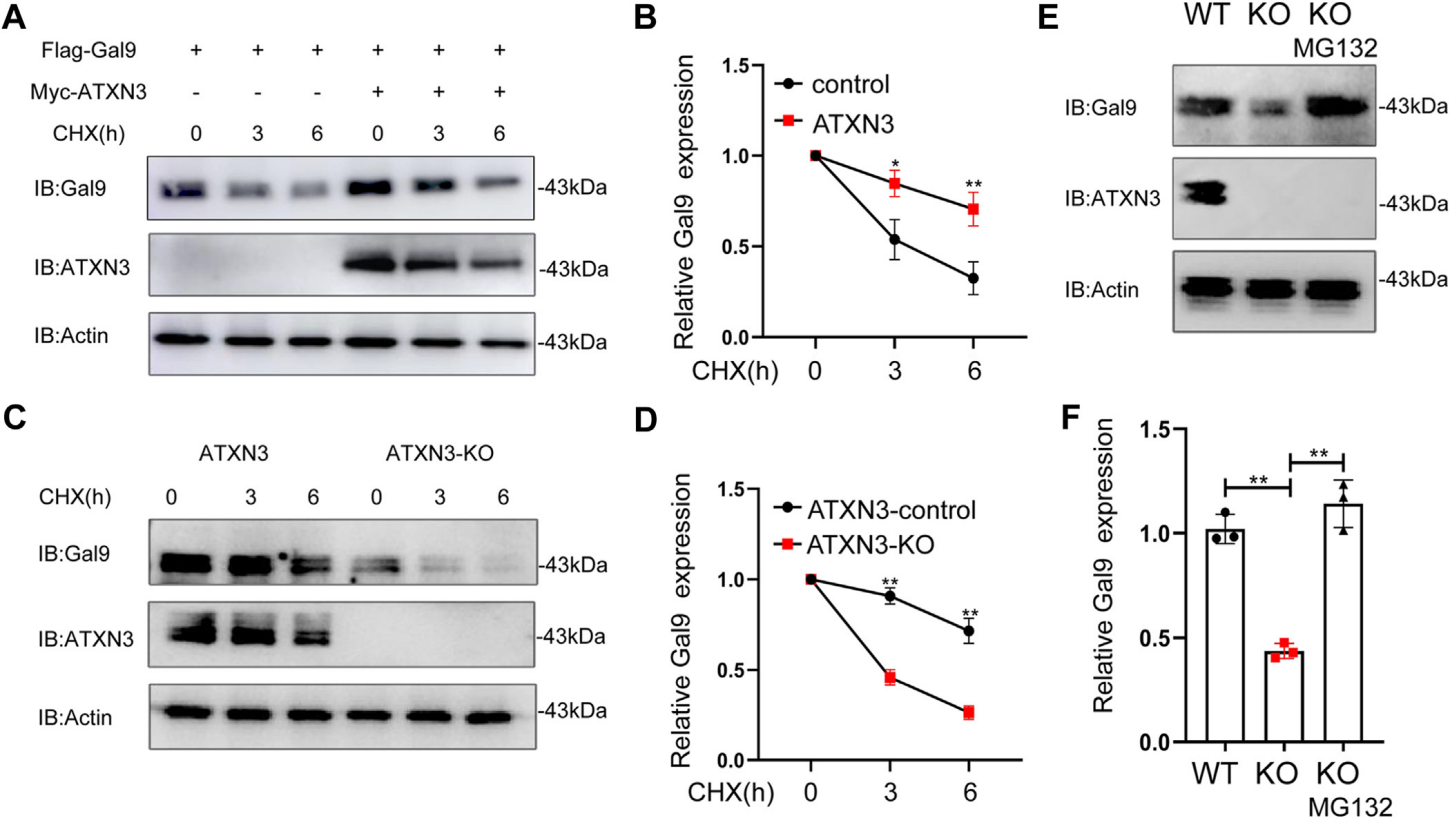
**ATXN3 protects Galectin-9 from proteasomal degradation.***A* and *B*, Galectin-9 was co-transfected with or without ATXN3 plasmids into HEK293T cells. The transfected cells were treated with CHX for different times. The protein levels of Galectin-9 (*top panel*) and ATXN3 (*middle panel*) were analyzed by Western blotting. Actin was used as a loading control (*bottom panel*). *C* and *D*, Immunoblot analysis of Galectin-9 protein stability in WT and ATXN3-KO HCT116 cells. *E* and *F*, HCT116 Cells were treated with or without MG132(40 μM) for 2 h, and the expression levels of Galectin-9, ATXN3, and Actin were analyzed by Western blotting in WT and ATXN3-KO HCT116 cell. *B* and *D*, two-tailed unpaired *t* test; *F*: Ordinary 1-way ANOVA. ∗*p* < 0.05, ∗∗*p* < 0.01, ∗∗∗*p* < 0.001.

In its role as a deubiquitinase, ATXN3 likely shields Galectin-9 from ubiquitination-induced protein degradation. We then performed pulse-chase experiments as recently reported (21, 22, 23) and demonstrated that the co-expression of ATXN3 extended the half-life of Galectin-9 (Fig. 3, *A* and *B*). Conversely, the absence of ATXN3 expedited the degradation of Galectin-9 in colon cancer cells (Fig. 3, *C* and *D*). Both the proteasome and lysosome pathways are implicated in ubiquitination-induced protein degradation. Nevertheless, the treatment of ATXN3 knockout colon cancer cells with the proteasomal inhibitor MG132 completely restored Galectin-9 protein expression (Fig. 3, *E* and *F*). These findings unequivocally indicate that ATXN3 operates as a Galectin-9-specific deubiquitinase, safeguarding Galectin-9 from ubiquitination-mediated proteasomal degradation.

### ATXN3 inhibits colon cancer growth through Galectin-9 stabilization

**Figure 4:**
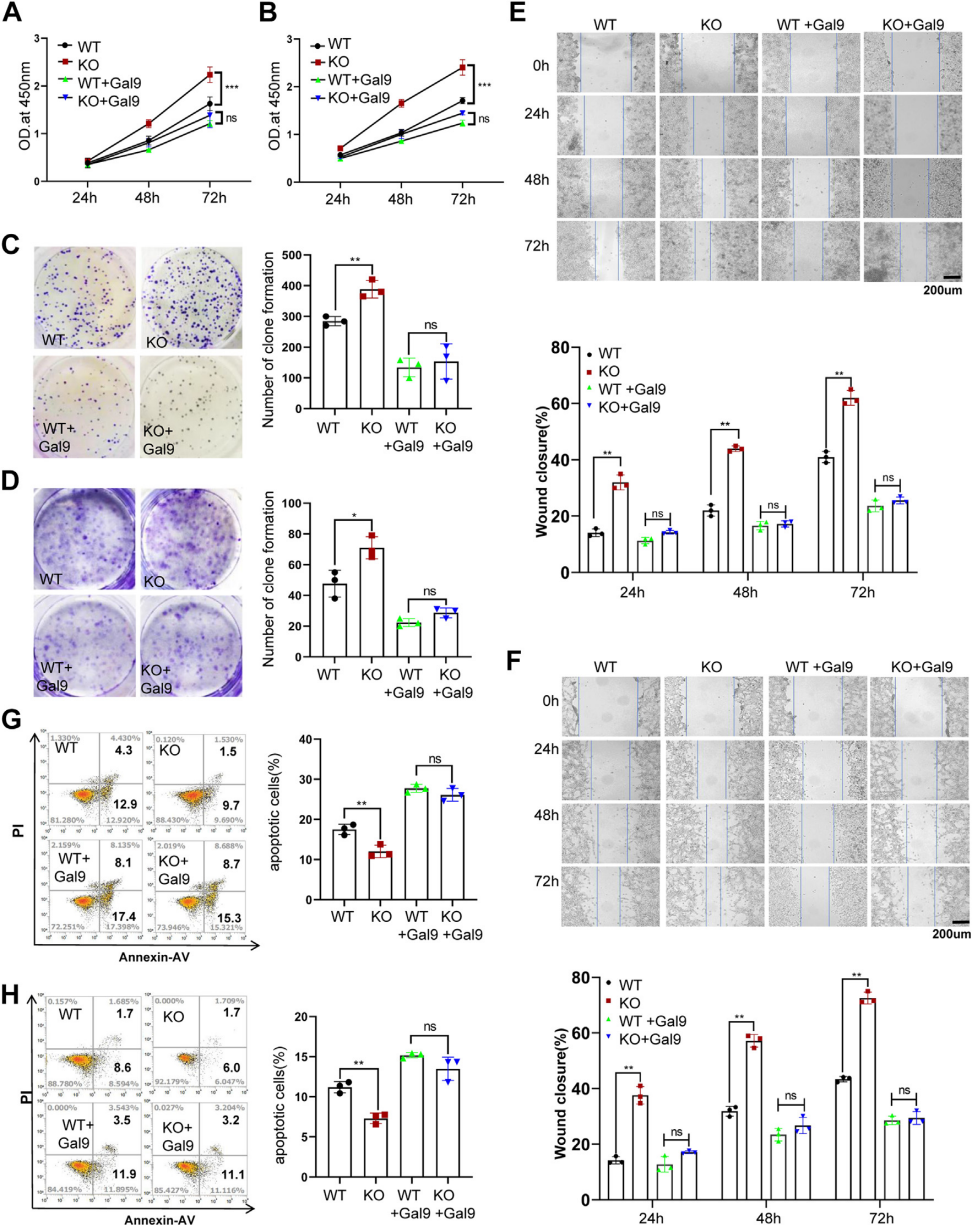
**ATXN3 inhibits colon cancer growth through Galectin-9.***A* and *B*, Galectin-9 was transiently transfected into WT and ATXN3-KO HCT116 (*A*)/MC38 (*B*) cells, and cell proliferation was detected using Cell Counting Kit-8 and quantified at 450 nm absorbance. *C* and *D*, colony numbers were measured in WT and ATXN3-KO HCT116 (*C*)/MC38 (*D*) cells while transient transfection of Galectin-9 was performed and colony numbers were measured, respectively. *E* and *F*, wound healing assays were conducted to investigate the effect of Galectin-9 overexpression on cell migration in WT and ATXN3-KO HCT116 (*E*)/MC38 (*F*) cell. Scale bar: 200 μm. (*G* and *H*) The apoptosis of Galectin-9 was determined by PI and Annexin V staining in WT and ATXN3-KO HCT116(E)/MC38(*F*) cell. *A*–*H*, ordinary 1-way ANOVA. ∗*p* < 0.05, ∗∗*p* < 0.01, ∗∗∗*p* < 0.001.

Galectin-9 is recognized for its tumor-suppressive roles, promoting cancer cell apoptosis and inhibiting cancer cell growth (20), suggesting a tumor-inhibitory role for the Galectin-9 deubiquitinase ATXN3. Indeed, the targeted deletion of ATXN3 in human colon cancer cells, leading to a substantial reduction in Galectin-9, markedly intensified both HCT116 and MC38 colon cancer cell proliferation (Fig. 4, *A* and *B*). Crucially, the restoration of Galectin-9 expression completely countered the increase in ATXN3-null cancer cell growth (Figs. 4, *A* and *B* and S2), unequivocally demonstrating that ATXN3 executes its tumor-suppressive functions through the upregulation of Galectin-9. Consistently, the loss of ATXN3 functions significantly enhanced the colony formation of HCT116 (Fig. 4*C*) and MC38 (Fig. 4*D*) colon cancer cells, which was entirely reversed by the expression of Galectin-9 (Figs. 4, *C* and *D* and S1). The Galectin-9-dependent tumor-suppressive functions of ATXN3 were further corroborated by a wound healing assay (Fig. 4, *E* and *F*). Additionally, flow cytometry analysis revealed a significant reduction in colon cancer cell death upon ATXN3 ablation, with Galectin-9 expression reversing the apoptosis of ATXN3-null colon cancer cells (Fig. 4, *G* and *H*). Collectively, these results affirm that ATXN3 functions as a tumor suppressor by enhancing Galectin-9-induced colon cancer cell death.

### Targeted ATXN3 deletion promotes colon cancer growth in mice

**Figure 5:**
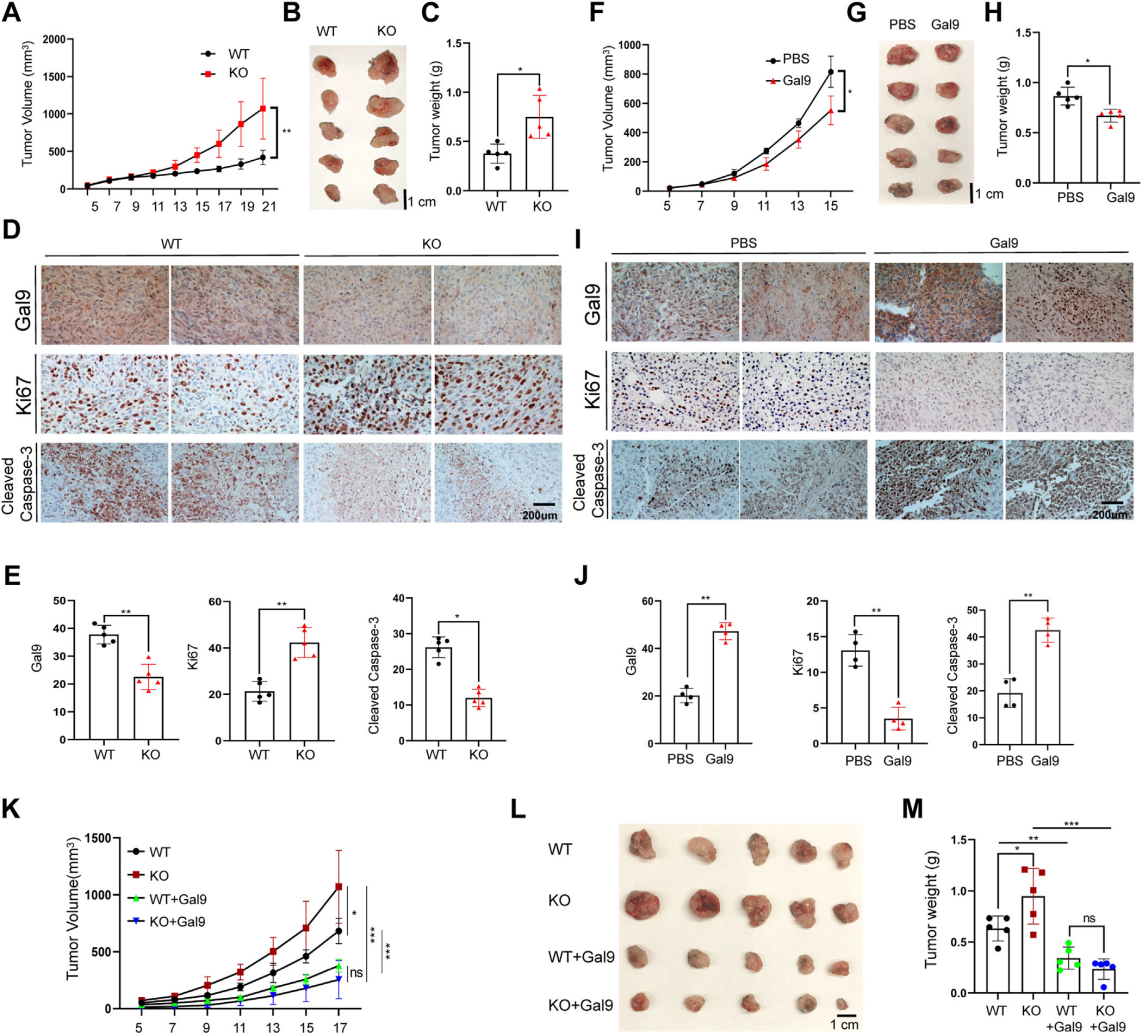
**Targeted ATXN3 deletion inhibits colon cancer growth in mice.***A*–*C*, WT or ATXN3-KO HCT116 cells were injected subcutaneously into RAG1 mutant mice (n = 10). Tumor growth curve (*A*), photograph (*B*), and weight (*C*) are shown. *D* and *E*, the protein expression of Galectin-9, Ki67, Cleaved Caspase-3 in (*B*) tumor was detected by IHC and measured relative expression by image J software(n = 5). Scale bar: 200 μm. *F*–*H*, HCT116 cells were injected subcutaneously into RAG1 mutant mice and treated with Galectin-9 recombinant protein (n = 10). Tumor growth curve (*F*), photograph (*G*), and weight (*H*) are shown. *I* and *J*, the protein expression of Galectin-9, Ki67, Cleaved Caspase-3 in (*G*) tumor was detected by IHC and measured relative expression by image J software(n = 4). Scale bar: 200 μm. *K*–*M*, tumor growth curve (*K*) of RAG1 mutant mice injected subcutaneously with WT or ATXN3-KO HCT116 cells and stabilize overexpression of Galectin-9 by lentivirus (n = 20). Tumor photograph (*L*) and tumor weight (*M*) are shown. *A*, *C*, *F*, *H*, *E*, and *J*, 2-tailed unpaired *t* test. ∗*p* < 0.05, ∗∗*p* < 0.01, ∗∗∗*p* < 0.001. L and M: ordinary 1-way ANOVA. ∗*p* < 0.05, ∗∗*p* < 0.01,∗∗∗*p* < 0.001.

Subsequently, we investigated whether genetic suppression of ATXN3 enhances colon cancer progression in mice. Given that ATXN3 modulates tumoral PD-L1 expression to evade antitumor immunosurveillance, we opted to utilize a xenograft colon cancer model to examine the impact of ATXN3 on cancer growth in immune-compromised mice. As anticipated, ATXN3 ablation led to a pronounced increase in HCT116 xenograft colon cancer growth in RAG1 mutant mice (Fig. 5, *A*–*C*). Further immunohistochemistry staining confirmed a significant reduction in Galectin-9 expression levels in the xenograft tumor tissues. Crucially, aligning with the heightened tumor growth, the deletion of ATXN3 dramatically increased the frequency of Ki-67^+^ proliferative cells (Fig. 5, *D* and *E*), indicating that ATXN3 reduced tumor progression by inhibiting Galectin-9 degradation, which consequently enhanced the Galectin-9-induced cancer cell apoptosis. To substantiate this concept, immunostaining detected a significant decrease in cleaved caspase-3 positive cells in ATXN3-null cancer tissue sections (Fig. 5, *D* and *E*). Conversely, the treatment of mice with recombinant Galectin-9 significantly inhibited colon cancer growth (Fig. 5, *F*–*H*). Consistently, Galectin-9 treatment resulted in the elevated colon cancer cell death and inhibited their growth as documented by the increased cleaved caspase-3 positive cells and reduced ki67^+^ cells. In addition, we also detected the elevated Galectin-9 levels in the tumor tissues from treated mice, possibly due to the accumulation of exogenous Galectin-9 (Fig. 5, *I* and *J*).

Subsequently, we established stable expression of Galectin-9 in ATXN3 wild-type and KO colon cancer cells to investigate whether reintroducing Galectin-9 expression could mitigate the heightened tumor progression observed upon ATXN3-targeted suppression. Flow cytometry analysis confirmed similar levels of Galectin-9 expression in the stably expressed ATXN3 WT and KO colon cancer cells (Fig. S2). As expected, the stable expression of Galectin-9 completely neutralized the increased tumor growth induced by ATXN3 suppression (Fig. 5, *K*–*M*), indicating that ATXN3 impedes colon cancer cell growth by stabilizing Galectin-9.

### ATXN3 protein expression is reduced and positively correlates with Galectin-9 in human colon cancer

**Figure 6:**
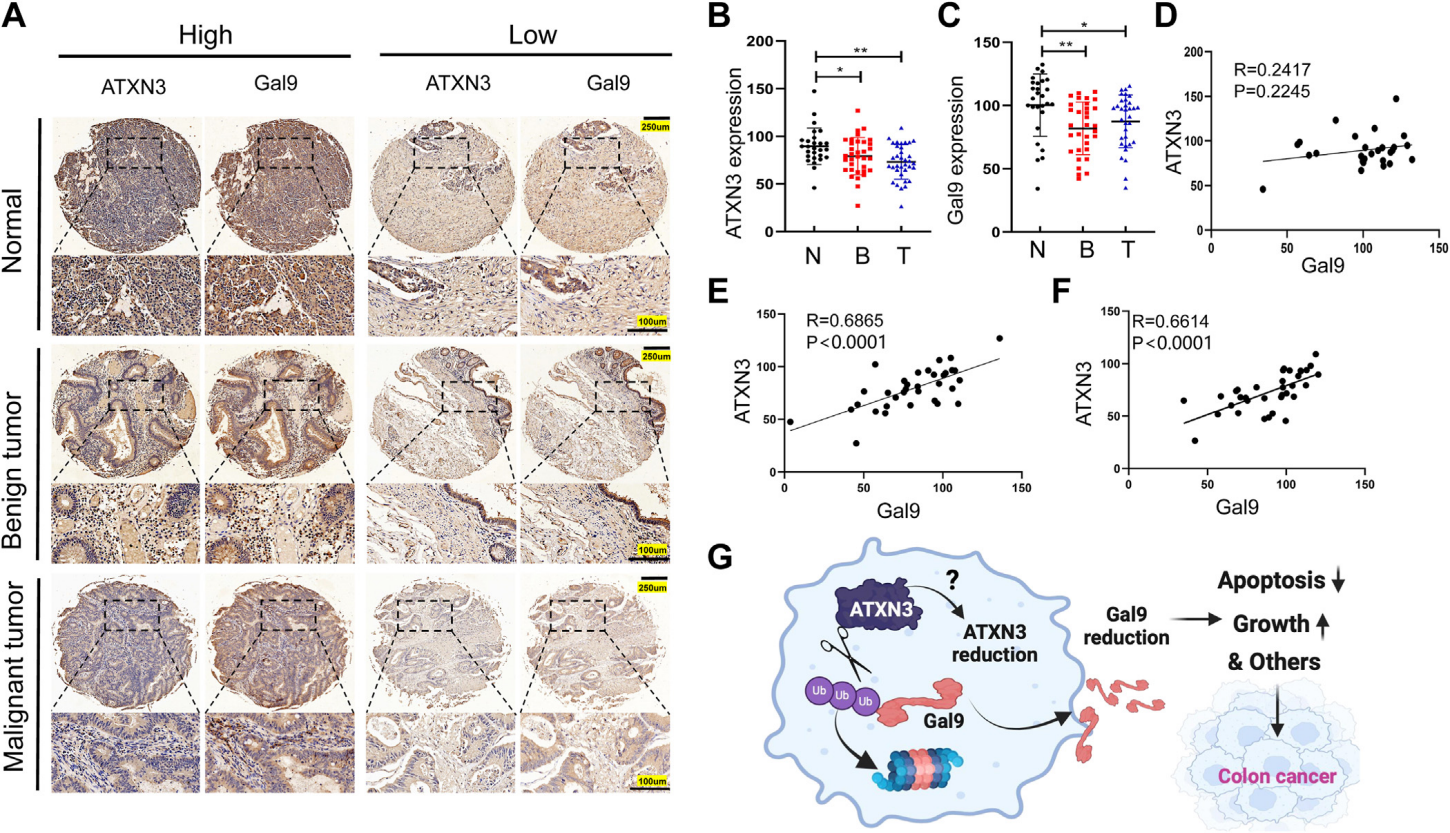
**ATXN3 protein expression is reduced and positively correlate with Galectin-9 in human colon cancer.***A*, representative images from immunohistochemical staining of ATXN3 and Galectin-9 in human colon adenocarcinoma. Scale bar: top row for 250 μm and bottom row for 100 μm. *B* and *C*, ATXN3 and Galectin-9 protein levels in tumor tissues compared with normal tissues in human colon adenocarcinoma patients (N: normal, n = 27; *B*: Benign tumor, n = 34; T: Malignant tumor, n = 34). *D*, correlation analysis of ATXN3 expression with Galectin-9 expression in normal patients (n = 27). *E*, correlation analysis of ATXN3 expression with Galectin-9 expression in benign tumor patients (n = 34). *F*, correlation analysis of ATXN3 expression with Galectin-9 expression in malignant tumor patients (n = 34). *G*, ATXN3 inhibits the proliferation of colon cancer by deubiquitinating Galectin-9. *B* and *C*: 2-tailed unpaired *t* test. *D*–*F*, Pearson’s correlation analysis. ∗∗*p* < 0.01, ∗∗∗*p* < 0.001.

Subsequently, we analyzed ATXN3 and Galectin-9 expression levels in human colon cancers. Immunohistochemical staining revealed variable protein expression levels for both ATXN3 and Galectin-9 in colon cancers (Figs. 6*A* and S3). Notably, quantitative analysis demonstrated a modest yet statistically significant reduction in both ATXN3 and Galectin-9 in malignant colon adenocarcinoma compared to adjacent normal colon tissues (Fig. 6, *B* and *C*). Both ATXN3 and Galectin-9 protein expression levels exhibited a similar decrease in benign colon adenoma compared to colon adenocarcinoma (Fig. 6, *B* and *C*), suggesting an early-stage involvement of ATXN3 and Galectin-9 reduction in colon cancer tumorigenesis. Intriguingly, a robust positive correlation between ATXN3 and Galectin-9 was observed in both colon adenomas and adenocarcinomas, as expected; however, this correlation was absent in adjacent healthy colon tissues (Fig. 6, *D*–*F*). These findings highlight a positive association between ATXN3 and Galectin-9 protein expressions in both benign and malignant tumors of the colon. On the molecular level, ATXN3 deubiquitinase acts on Galectin-9 as an endogenous substrate. The reduction of ATXN3 results in the degradation of Galectin-9 protein, thereby impeding Galectin-9-induced apoptosis and promoting the progression of cancer.

## Discussion

Our study establishes ATXN3 as the inaugural Galectin-9-specific deubiquitinase and brings to light a tumor-suppressive role of ATXN3 in human colon cancer. This conclusion is supported by the following findings: First, targeted ATXN3 deletion markedly diminished Galectin-9 protein levels without influencing its mRNA expression in human colon cancers; Second, ATXN3 interacts with and inhibits Galectin-9 ubiquitination, thereby shielding Galectin-9 from ubiquitination-induced proteasome degradation; Third, ATXN3 ablation heightened cell growth and suppressed apoptosis in colon cancers due to the decreased expression of Galectin-9; Last but not least, immunohistochemistry staining revealed a significant reduction in both ATXN3 and Galectin-9 protein expression, along with a positive correlation between them in human colon cancer.

Contrary to current studies highlighting the tumor-promoting effects of ATXN3, our research uncovers a tumor-suppressive role for ATXN3 in colon cancers, suggesting that ATXN3 functions as a complex regulator in tumorigenesis, possibly exhibiting cancer-type-specific behaviors. In alignment with our findings, it has been demonstrated that the oncogenic microRNA miR-25, frequently elevated in various human cancers, targets ATXN3 and suppresses its protein expression, thereby promoting colon cancer progression (24). Our study further reveals that ATXN3 exerts its tumor-suppressive functions by safeguarding Galectin-9, a carbohydrate-binding protein known for inducing apoptosis and inhibiting the proliferation of human cancer cells (20), from ubiquitination-mediated proteasomal degradation. Investigating whether the oncogenic miR-25 inhibits Galectin-9 in colon cancers presents an intriguing avenue for future research. Furthermore, Galectin-9 has been recognized as a crucial positive regulator of mucosal immunity in combatting gut inflammation caused by the microbiome through the Th17-IgA axis (25), establishing an additional connection for ATXN3 in suppressing inflammation-induced colon cancer. Apart from its role in impeding cancer cell growth and inducing apoptosis, Galectin-9 is well-known for its immune-suppressive properties, fostering immune-suppressive tumor microenvironments by binding to checkpoint receptors such as PD-1 and Tim-3 (26). Therefore, the regulatory functions of ATXN3 on Galectin-9 in tumorigenesis are intricate. Future studies using both immune functional and immunocompromised (this study) mice to dissect the tumor intrinsic and extrinsic contributions of ATXN3 in colon cancer pathogenesis are needed. Nevertheless, given ATXN3's dual roles as both oncogenic and tumor-suppressive, prudence is essential in the development of ATXN3-targeting strategies for antitumor therapy.

Galectins constitute a protein family that selectively binds to specific glycans, deciphering information encoded within the glycome. Galectin-9, a distinctive member of this family, features two non-homologous carbohydrate recognition domains connected by a variable-length linker peptide sequence, resulting in isoforms with distinct properties and functions in various physiological and pathological contexts, including development, immune responses, neoplastic transformations, and metastasis (27). While the transcriptional regulation of the Galectin-9 gene has been extensively studied, information regarding its posttranslational modifications is lacking. Kinome analysis of breast cancer cells has suggested that Galectin-9 can undergo phosphorylation (28). A comprehensive profiling of protein ubiquitination sites in mouse cells has identified lysine residue 87 of Galectin-9 as a ubiquitination site (29). However, the E3 ubiquitin ligase responsible for catalyzing Galectin-9 ubiquitination remains unidentified. In our research, we identified ATXN3 as the inaugural Galectin-9-specific deubiquitinase, shielding it from ubiquitination-mediated proteasomal degradation. Therefore, a strong positive correlation between ATXN3 and Galectin-9 protein in human colon cancer tissues was detected. However, a positive correlation between ATXN3 and Galectin-9 protein expression was not detected in adjacent healthy colon tissues. One possibility is due to a relatively smaller sample size. In addition, there could be a stronger transcriptional regulation of either genes or both in normal tissues than in cancers, which may override their protein correlation. Future studies are needed to precisely dissect the underlying molecular mechanisms in regulating ATXN3 and Galectin-9 expression under biological conditions *versus* cancer. In addition, USP9X has been recognized as a Galectin-9-interacting deubiquitinase during lysosomal damage (30), but its involvement in regulating Galectin-9 protein ubiquitination remains unclear.

## Experimental procedures

### Animal studies

C57BL/6J-Rag1 mice were bred and maintained at the Northwestern University mouse facility under specific pathogen-free conditions, keeping plenty of food and water. All figures presented in this study represent experiments conducted using mice that were 6 to 8 weeks old. For the syngeneic mouse tumor models, a total of 5 × 10^5^ to 1 × 10^6^ cells suspended in 100 μl of PBS were injected subcutaneously into the right flank of each mouse. Tumor measurements began on day 5 following tumor cell implantation and were subsequently recorded every 2 days using a digital caliper to measure the length and width. Tumor volume was calculated using the formula: volume = (width^2^) × length/2. None of the tumors reached a volume exceeding 2000 mm^3^ throughout the entire duration of the study. In order to achieve *in vivo* overexpression of the recombinant protein Galectin-9, mice were administered intraperitoneal injections of Galectin-9 recombinant protein (R&D Systems) every 2 days, with an average dosage of 1 μg per injection. All animal experiments followed the Northwestern University IACUC approved protocol: IS00015611.

### Cell culture, transfection, generating stable cell line, and cell treatment

HEK293 cell (ATCC, CRL-3216), HCT116 (ATCC, CCL-247) cells, and MC38 (obtained from Bin Zhang’s lab at Northwestern University) cells were cultured in Dulbecco's Modified Eagle Medium (DMEM) supplemented with 10% fetal bovine serum. CT26 (ATCC, CRL-2638) cells were maintained in RPMI 1640 medium supplemented with 10% FBS. Transfections were carried out using Lipofectamine 3000 (Invitrogen, Cat#: L3000-150) following the manufacturer's instructions. After 24- or 48-h post-transfection, cells were harvested and subjected to various assays. For ATXN3 gene knockout, cells are first transfected with CRISPR plasmids and were selected in the presence of puromycin (MCE, Cat#: HY-B1743Aa) for a minimum of 2 days to establish stable cell lines. The comprehensive list of all primers employed in the study is provided in Table S2. In cell degradation experiments, the transfected HEK293 cells or HCT116 cells were treated with cycloheximide (CHX) (Cell Signaling Technology, Cat#: 2112) for different durations of time.

### Plasmids and other reagents

The Myc-ATXN3 plasmid (Cat#: RC218923) was acquired from Origene. The Flag-Galectin-9-human plasmid (Cat#: p18073) was obtained from Wuhan Miaoling Biology in China. Mouse Galectin-9 gene fragments were amplified through PCR using complementary DNA (cDNA) derived from the MC38 cell line and subsequently cloned into the pCMV-Myc vector (Clontech) to create Galectin-9-mouse plasmid. The Myc-Galectin-9 (1–190aa), (190–355aa), and (146–355aa) truncations were created by introducing stop codons using the Q5 Site-Directed Mutagenesis Kit (Cat#E0054S).

### Flow cytometry analysis of Galectin-9

For cultured cell lines, cells were enzymatically detached using Accutase solution and subsequently harvested through centrifugation at 1500 rpm for 5 min. The harvested cells were subjected to staining procedures, incorporating a fixable viability dye (eBioscience eFluor 450) and an APC-conjugated Galectin-9 antibody (BioLegend, catalog 137911 for mouse cells and 348,907 for human cells) at 4 °C for 30 min in the absence of light. Following staining, the cells were thoroughly washed with FACS Buffer. The cells were analyzed using a BD flow cytometer, and the acquired data were subjected to analysis using FlowJo software.

### Generation of stable Galectin-9-overexpressing cell lines

To induce stable overexpression of Galectin-9 in cells, we initiated the process by subcloning the coding sequence of Galectin-9 into a pLV lentiviral vector as we reported (31, 32). This subcloning procedure involved the use of two restriction enzymes, namely BamHI and EcoRI, following a double digest protocol. As a negative control, an empty vector was employed. Subsequently, the Galectin-9 expression construct was cotransfected with packaging plasmids into HEK293T cells using the jetPRIME Transfection reagent. Following a 48-h incubation duration, the lentiviral bearing the Galectin-9 expression were methodically collected. These lentiviruses were then utilized to infect the target cells. After a 2-days incubation period, the cells were subjected to analysis using a BD flow cytometer to confirm the stable overexpression of Galectin-9.

### Co-immunoprecipitation and Western blotting

Co-immunoprecipitation and western blotting were performed as we recently reported (3, 4). The cells were rinsed with ice-cold PBS twice and subsequently lysed in RIPA lysis buffer containing a protease inhibitor. The lysates were incubated on ice for 30 min, followed by centrifugation at 14,000 rpm for 15 min. Supernatants were pre-cleared with protein-G sepharose (GE Healthcare, Cat#: 17-0618-02) through three consecutive rounds of washing, each lasting 20 min. The pre-cleared supernatants were then subjected to immunoprecipitation using the specified antibodies. This incubation took place for 2 to 4 h on a shaker in a cold room, followed by the addition of 60 μl of protein-G sepharose beads overnight. The resulting bead complexes underwent five washes by PBS and were subsequently boiled with 60 μl of 2 × loading buffer for 7 min. The proteins were separated on 8 to 15% SDS-PAGE gels and transferred onto nitrocellulose membranes. The membranes were blocked in 5% fat-free dried milk in Tris-buffered saline with 0.5% Tween 20 (TBST) for 1 h. After blocking, the membranes were incubated with the appropriate primary antibodies overnight at 4 °C. Following the incubation with the primary antibody, membranes underwent a triple wash procedure using Tris-buffered saline with Tween 20 and incubated with horseradish peroxidase (HRP)-conjugated secondary antibodies (EMD Millipore Corp, goat anti-rabbit IgG antibody, HRP conjugate, Cat#: 12-348; goat anti-mouse IgG antibody, HRP conjugate, Cat#: 12-349) for 1.5 h. Subsequently, the membranes underwent additional TBST washes, and the signals were visualized using enhanced chemiluminescence substrate (Thermo Scientific, Cat#: 34577) and quantified using Bio-Rad Image software. The primary antibodies employed in this study were as follows: ATXN3 (Protein tech, Cat#: 67057-1-lg, 13505-1-AP), Galectin-9 (Abcam, Cat#: ab69630-1001), Galectin-9 (Protein tech, Cat#: 17938-1-AP), β-actin (Protein tech, Cat#: 66009-1-lg), Flag-tag (Sigma, Cat#: F1804), Myc-tag (Cell Signaling Technology, Cat#: 2278S), Ubiquitin (Cell Signaling Technology, Cat#: 3936S), and HA HRP (Cell Signaling Technology, Cat#: 2999S).

### Real-time quantitative PCR with reverse transcription

Total RNA extraction was carried out utilizing the RNeasy Micro Kit (Qiagen, Cat#: 74106), followed by reverse transcription using the qScript cDNA synthesis kit (Quanta Bioscience, Cat#: 84003). Subsequent to cDNA synthesis, quantitative PCR was conducted employing the qScript cDNA Synthesis kit (Quanta Biosciences, Cat#: 95047-100). The quantification of mRNA levels was performed utilizing the ΔCt method, and subsequent normalization was executed relative to GAPDH or β-actin. The comprehensive list of all primers employed in the study is provided in Table S1.

### Cell proliferation assay

*In vitro* cell proliferation was assessed employing the Cell Counting Kit-8 (Abcam, ab228554). 2000 to 3000 HCT116/MC38 cells were seeded in a 96-well plate with DMEM supplemented with 10% fetal bovine serum. After 12 to 24 h, 10 μl of WST-8 reagent was introduced into each well, followed by an incubation period of 1 to 2 h. Subsequently, the absorbance at 450 nm was quantified using a microplate reader.

### Cell colony formation experiment

As we recently reported (33), a total of 1000 HCT116/MC38 cells were seeded in a 6-well plate containing DMEM supplemented with 10% fetal bovine serum. Subsequently, the cells were allowed to incubate for a duration of 7 to 14 days. Following the incubation period, the cells were fixed by the addition of 1 ml of methanol per well for 30 min. Post-fixation, the cells were stained with 0.1% crystal violet for 10 min. The excess liquid was aspirated, and the cells were rinsed with running water before being allowed to air-dry naturally. The enumeration of colonies was performed utilizing Image-J software.

### Apoptosis assays

Cells were enzymatically detached using Accutase solution (Corning, Cat#: 25-058-CI), and the resulting cell suspension was collected by centrifugation at 1200 rpm for 5 min. The cells were then washed twice with cold Annexin V binding buffer. Subsequently, the cells were stained in Annexin V binding buffer (Biolegend, Cat#: 42201) with APC-Annexin V (1:100, Biolegend, Cat#: 640-920) and propidium iodide (PI) (1:1000, Biolegend, Cat#: 79997) antibodies. Following staining, the cells were subjected to flow cytometric analysis using a BD flow cytometer, and the acquired data were analyzed using FlowJo software.

### Wound-healing assay

HCT116/MC38 cells were cultured in 6-well plates and subsequently transfected with the designated plasmids. Following a 24-h post-transfection period, the cell monolayer was subjected to mechanical injury by carefully scratching it with a sterile 20 μl pipette tip. Subsequently, images of the scratches were captured at specific time points: 0, 24, 48, and 72 h. The analysis of scratch areas was performed using Image-J software.

### Immunohistochemical staining of colon cancer tissue microarrays and mouse tumor

Tumor tissue microarrays, sourced from Bioaitech Co Ltd (Cat#: DC-Col11067), encompass a total of 27 normal colonic tissue cases and 69 colon cancer cases. Paraffin-embedded human tissue microarrays underwent a series of procedures, including deparaffinization, rehydration, heat-induced antigen retrieval, and blocking in goat serum blocking solution at room temperature for 30 min. Subsequently, the arrays were incubated with primary antibodies overnight at 4 °C. The primary antibodies employed for immunohistochemistry (IHC) were directed against Galectin-9 (1:100, Proteintech, Cat#: 17938-1-AP) and ATXN3 (1:200, Proteintech, Cat#: 13505-1-AP). On the following day, the sections underwent washing and were then incubated with biotin-conjugated secondary antibodies for 1 h at room temperature, followed by a DAB chromogenic reaction. Nuclei were counterstained using hematoxylin stain solution, and the specimens were mounted. Imaging was conducted with a Nikon microscope, and subsequent analysis was performed using Aipathwell software. The percentage of positive area represents the proportion of positive area relative to the entire tissue area. Other antibodies are as follows: Ki-67(1:200, Cell Signaling Technology, Cat#: 12202S); NME1(1:100, Cell Signaling Technology, Cat#: 3345S); Cleaved Caspase-3(1:400, Cell Signaling Technology, Cat#: 9661S).

### Statistical analyses

Statistical analysis was conducted utilizing GraphPad Prism 8 software. Group comparisons were executed through unpaired two-tailed Student’s *t* test unless otherwise specified, with statistical significance set at *p* < 0.05. Pearson’s correlation analysis was employed to assess the correlation between two variables. The findings are reported as the mean ± SD derived from three distinct experimental iterations; statistical significance is denoted as follows: ∗*p* < 0.05, ∗∗*p* < 0.01, ∗∗∗*p* < 0.001.

## Data availability

Values for all data points found in graphs can be found in the supporting data values file. All raw, uncropped Western blots are available as supplemental material. Additional details regarding data and protocols that support the findings of this study are available from the corresponding author upon request.

## Supporting information

This article contains supporting information.

## Conflict of interest

The authors declare that they have no known competing financial interests or personal relationships that could have appeared to influence the work reported in this paper.

## References

[1] Wang X.M., Yang C., Zhao Y., Xu Z.G., Yang W., Wang P. (2020). The deubiquitinase USP25 supports colonic inflammation and bacterial infection and promotes colorectal cancer. Nat. Cancer.

[2] Lin Z., Yang H., Tan C., Li J., Liu Z., Quan Q. (2013). USP10 antagonizes c-Myc transcriptional activation through SIRT6 stabilization to suppress tumor formation. Cell Rep..

[3] Lin Z., Tan C., Qiu Q., Kong S., Yang H., Zhao F. (2015). Ubiquitin-specific protease 22 is a deubiquitinase of CCNB1. Cell Discov..

[4] Lin Z., Yang H., Kong Q., Li J., Lee S.M., Gao B. (2012). USP22 antagonizes p53 transcriptional activation by deubiquitinating Sirt1 to suppress cell apoptosis and is required for mouse embryonic development. Mol. Cell.

[5] Popov N., Wanzel M., Madiredjo M., Zhang D., Beijersbergen R., Bernards R. (2007). The ubiquitin-specific protease USP28 is required for MYC stability. Nat. Cell Biol..

[6] Iliopoulos D., Jaeger S.A., Hirsch H.A., Bulyk M.L., Struhl K. (2010). STAT3 activation of miR-21 and miR-181b-1 via PTEN and CYLD are part of the epigenetic switch linking inflammation to cancer. Mol. Cell.

[7] Gennaro V.J., Stanek T.J., Peck A.R., Sun Y., Wang F., Qie S. (2018). Control of CCND1 ubiquitylation by the catalytic SAGA subunit USP22 is essential for cell cycle progression through G1 in cancer cells. Proc. Natl. Acad. Sci. U. S. A..

[8] McFarlane C., Kelvin A.A., de la Vega M., Govender U., Scott C.J., Burrows J.F. (2010). The deubiquitinating enzyme USP17 is highly expressed in tumor biopsies, is cell cycle regulated, and is required for G1-S progression. Cancer Res..

[9] Song J., Liu T., Yin Y., Zhao W., Lin Z., Yin Y. (2021). The deubiquitinase OTUD1 enhances iron transport and potentiates host antitumor immunity. EMBO Rep..

[10] Li J.J., Wang J.H., Tian T., Liu J., Zheng Y.Q., Mo H.Y. (2023). The liver microenvironment orchestrates FGL1-mediated immune escape and progression of metastatic colorectal cancer. Nat. Commun..

[11] Zhang P., Li C., Li H., Yuan L., Dai H., Peng Z. (2020). Ubiquitin ligase CHIP regulates OTUD3 stability and suppresses tumour metastasis in lung Cancer. Cell Death Differ..

[12] Yu K., Guo Y.Y., Liuyu T., Wang P., Zhang Z.D., Lin D. (2023). The deubiquitinase OTUD4 inhibits the expression of antimicrobial peptides in Paneth cells to support intestinal inflammation and bacterial infection. Cell Insight.

[13] Khan O.M., Carvalho J., Spencer-Dene B., Mitter R., Frith D., Snijders A.P. (2018). The deubiquitinase USP9X regulates FBW7 stability and suppresses colorectal Cancer. J. Clin. Invest..

[14] Switonski P.M., Fiszer A., Kazmierska K., Kurpisz M., Krzyzosiak W.J., Figiel M. (2011). Mouse ataxin-3 functional knock-out model. Neuromolecular Med..

[15] Sacco J.J., Yau T.Y., Darling S., Patel V., Liu H., Urbe S. (2014). The deubiquitylase Ataxin-3 restricts PTEN transcription in lung cancer cells. Oncogene.

[16] Zou H., Chen H., Zhou Z., Wan Y., Liu Z. (2019). ATXN3 promotes breast cancer metastasis by deubiquitinating KLF4. Cancer Lett..

[17] Wu L., Ou Z., Liu P., Zhao C., Tong S., Wang R. (2023). ATXN3 promotes prostate cancer progression by stabilizing YAP. Cell Commun. Signal..

[18] Wang S., Liu K., Han X., Cheng Y., Zhao E., Brat D.J. (2023). ATXN3 deubiquitinates YAP1 to promote tumor growth. Am. J. Cancer Res..

[19] Wang S., Iyer R., Han X., Wei J., Li N., Cheng Y. (2023). CRISPR screening identifies the deubiquitylase ATXN3 as a PD-L1-positive regulator for tumor immune evasion. J. Clin. Invest..

[20] Morishita A., Nomura K., Tani J., Fujita K., Iwama H., Takuma K. (2021). Galectin-9 suppresses the tumor growth of colon cancer in vitro and in vivo. Oncol. Rep..

[21] Wei J., Chen L., Li F., Yuan Y., Wang Y., Xia W. (2018). HRD1-ERAD controls production of the hepatokine FGF21 through CREBH polyubiquitination. EMBO J..

[22] Wei J., Harada B.T., Lu D., Ma R., Gao B., Xu Y. (2021). HRD1-mediated METTL14 degradation regulates m(6)A mRNA modification to suppress ER proteotoxic liver disease. Mol. Cell.

[23] Wei J., Yuan Y., Chen L., Xu Y., Zhang Y., Wang Y. (2018). ER-associated ubiquitin ligase HRD1 programs liver metabolism by targeting multiple metabolic enzymes. Nat. Commun..

[24] Li D., Zhang T., Lai J., Zhang J., Wang T., Ling Y. (2019). MicroRNA-25/ATXN3 interaction regulates human colon cancer cell growth and migration. Mol. Med. Rep..

[25] Liang C.C., Li C.S., Weng I.C., Chen H.Y., Lu H.H., Huang C.C. (2018). Galectin-9 is critical for mucosal Adaptive immunity through the T Helper 17-IgA Axis. Am. J. Pathol..

[26] Quilbe A., Mustapha R., Duchene B., Kumar A., Werkmeister E., Leteurtre E. (2023). A novel anti-galectin-9 immunotherapy limits the early progression of pancreatic neoplastic lesions in transgenic mice. Front. Immunol..

[27] John S., Mishra R. (2016). Galectin-9: from cell biology to complex disease dynamics. J. Biosci..

[28] Mertins P., Mani D.R., Ruggles K.V., Gillette M.A., Clauser K.R., Wang P. (2016). Proteogenomics connects somatic mutations to signalling in breast cancer. Nature.

[29] Wagner S.A., Beli P., Weinert B.T., Scholz C., Kelstrup C.D., Young C. (2012). Proteomic analyses reveal divergent ubiquitylation site patterns in murine tissues. Mol. Cell. Proteomics.

[30] Jia J., Bissa B., Brecht L., Allers L., Choi S.W., Gu Y. (2020). AMPK, a regulator of metabolism and Autophagy, is activated by lysosomal damage via a novel galectin-directed ubiquitin signal transduction system. Mol. Cell.

[31] Lee S.M., Gao B., Fang D. (2008). FoxP3 maintains Treg unresponsiveness by selectively inhibiting the promoter DNA-binding activity of AP-1. Blood.

[32] Cortez J.T., Montauti E., Shifrut E., Gatchalian J., Zhang Y., Shaked O. (2020). CRISPR screen in regulatory T cells reveals modulators of Foxp3. Nature.

[33] Liu K., Gao Q., Jia Y., Wei J., Chaudhuri S., Wang S. (2023). Ubiquitin-specific peptidase 22 controls integrin-dependent cancer cell stemness and metastasis. Res. Sq..

