# Supplementary material for: ATXN3 functions as a tumor suppressor through potentiating galectin-9-mediated apoptosis in human colon adenocarcinoma
Source: J Biol Chem. 2024 May 28;300(7):107415. doi: 10.1016/j.jbc.2024.107415 (PMC11254720; doi:10.1016/j.jbc.2024.107415)
Supplement: Supporting Information [file mmc1.docx]

**ATXN3 functions as a tumor suppresser through potentiating Galectin-9-mediated apoptosis in** **human colon adenocarcinoma**

Yang Cheng^1^, Shengnan Wang^1^, Qiong Gao^1^ and Deyu Fang^1, 2, 3 *^

^1^Department of Pathology, ^2^Robert H. Lurie Comprehensive Cancer Center, and ^3^Center for Human Immunology, Northwestern University Feinberg School of Medicine, Chicago, IL, USA 60611.

**Supplemental figures 1 & 2 & 3 and legends**

**Supplemental tables 1 & 2**


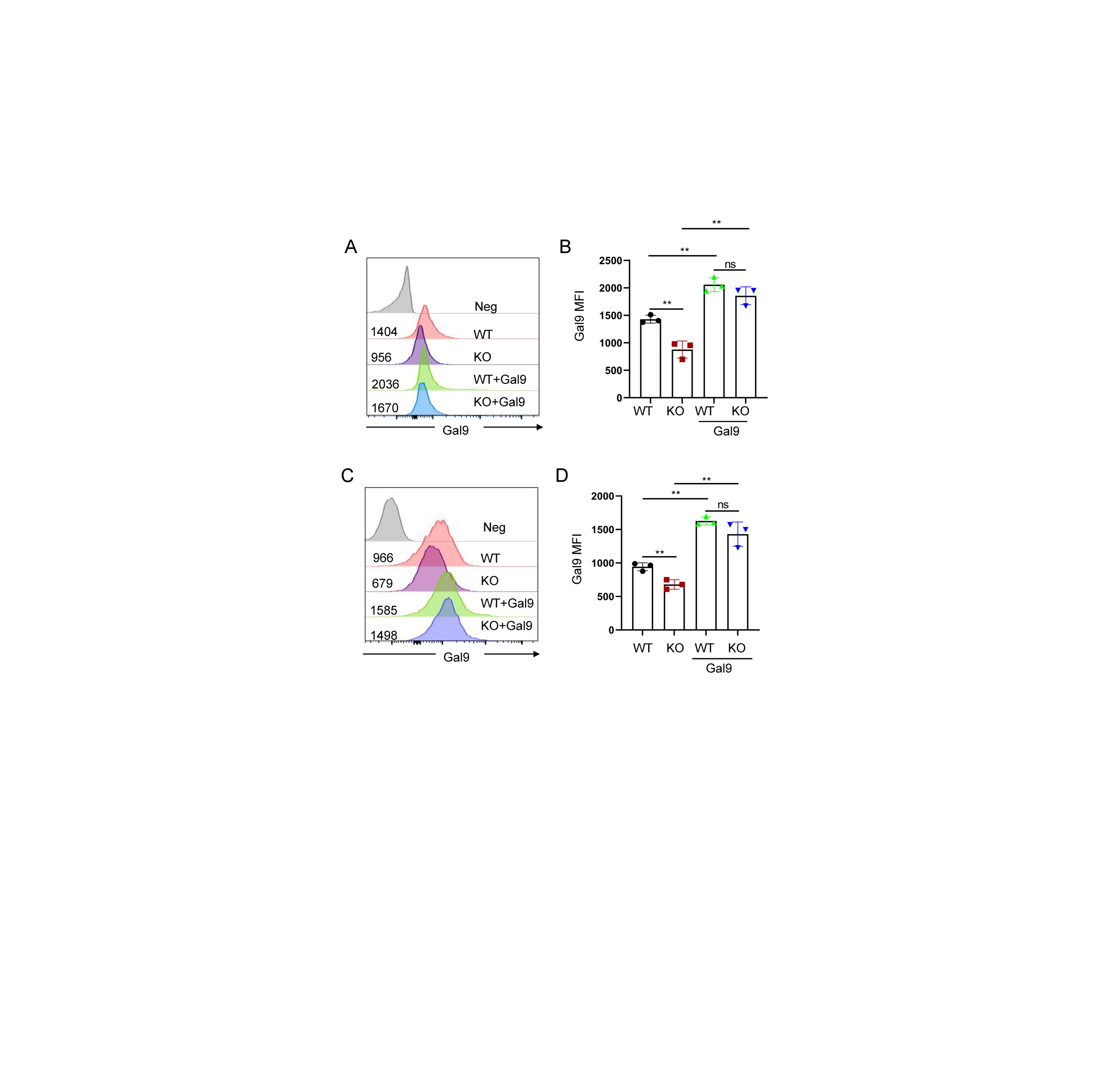


**Figure S1. Gal9 overexpression was verified by flow.**

(A & B) Gal9 was transfected into HCT116 cells instantaneously and checked the Gal9 expression by flow cytometry. (C & D) Gal9 was transfected into MC38 cells instantaneously and checked the Gal9 expression by flow cytometry. B and D: ordinary 1-way ANOVA. *P < 0.05, **P < 0.01,***P < 0.001.


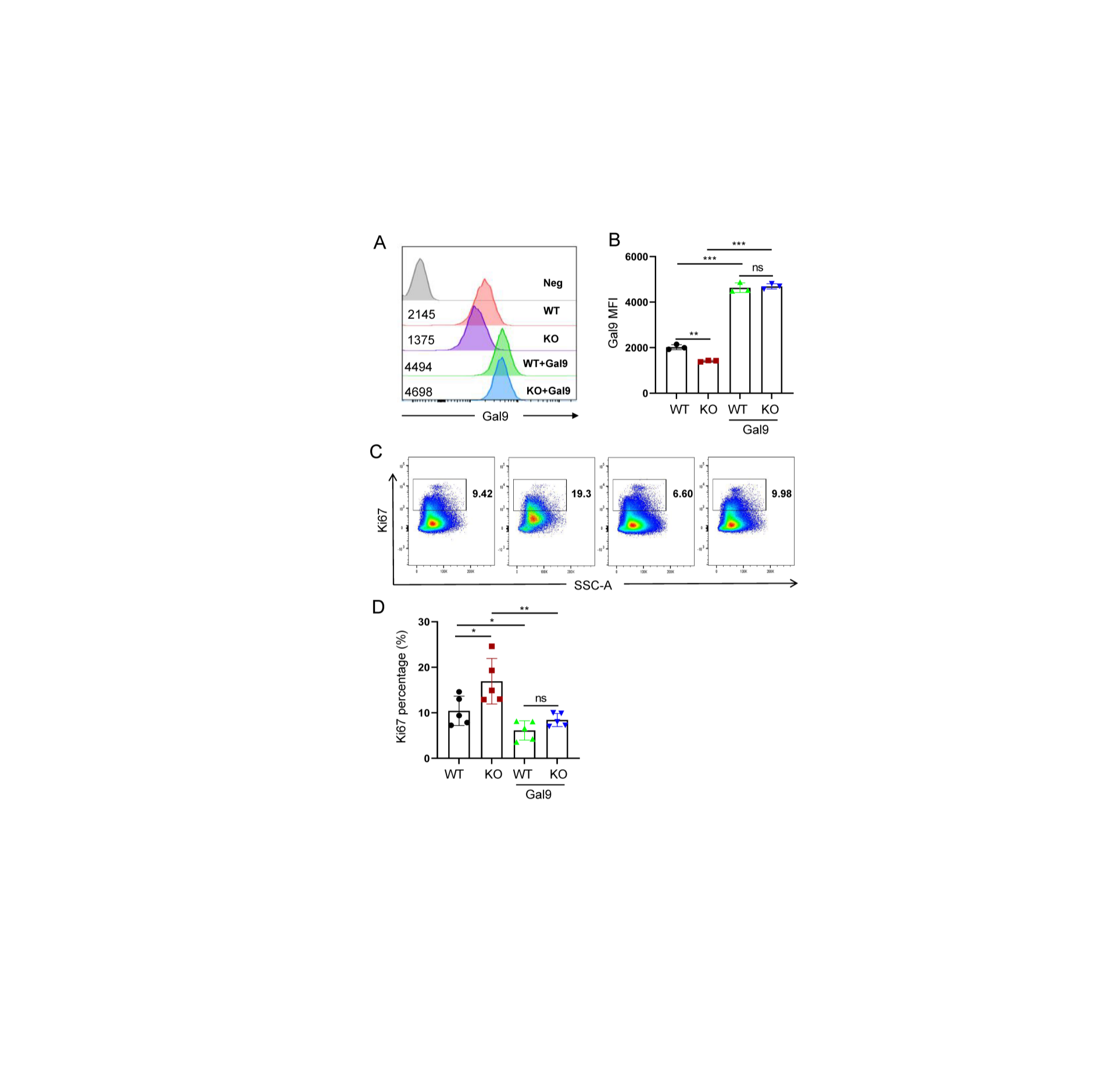


**Figure S2. ATXN3 regulates colon cancer cell proliferation through Galectin-9.**

(A & B) Gal9 was stably expressed into HCT116 cells by lentivirus and measured the expression by flow cytometry. (C a& D) The percentage of Ki67 was measured in RAG1 mutant mice tumor by flow cytometry. B and D: ordinary 1-way ANOVA. *P < 0.05, **P < 0.01,***P < 0.001.


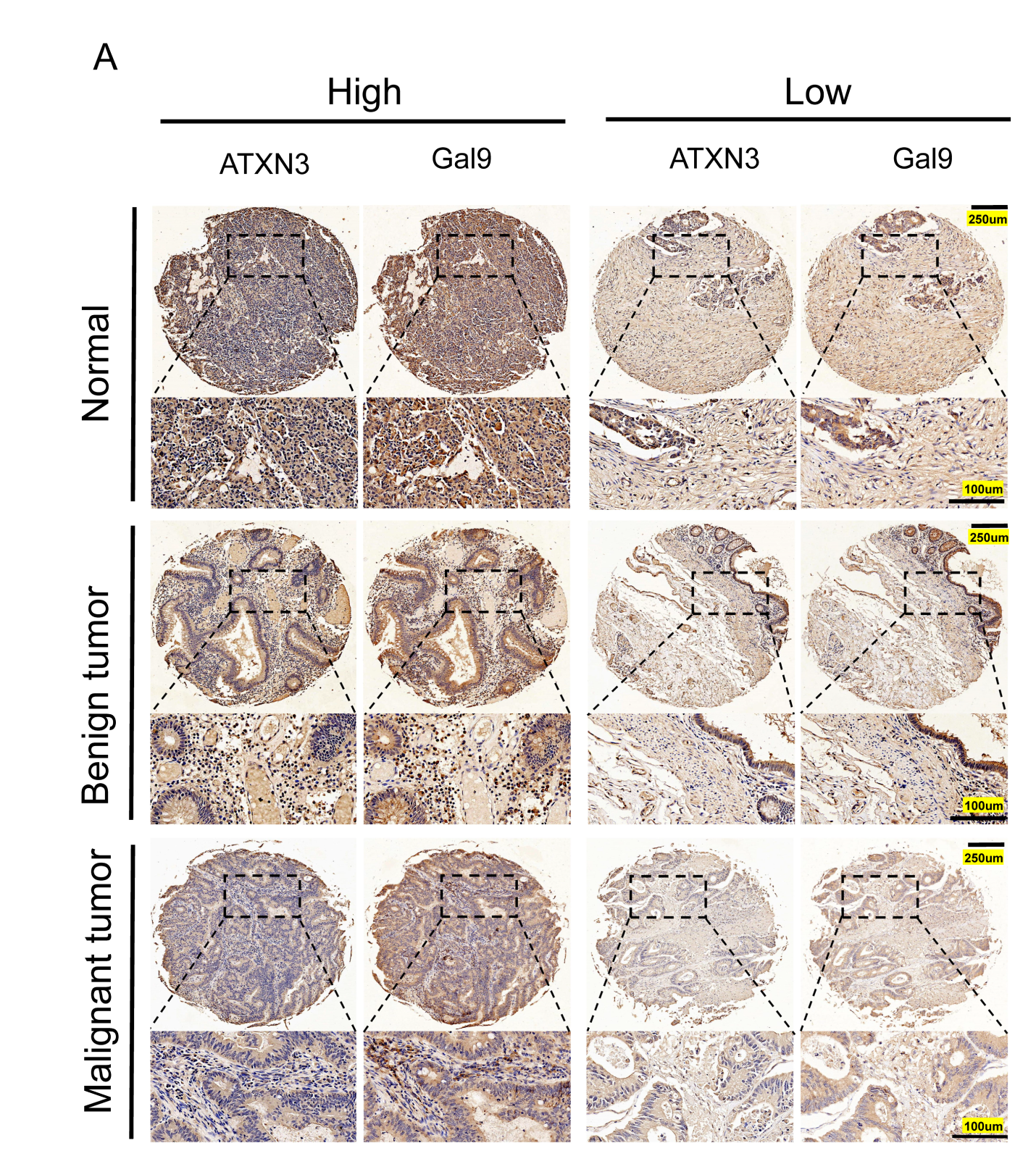


**Figure S3. Images in Fig. 6A with high resolution showing that ATXN3 protein expression is reduced and positively correlate with Galectin-9 in human colon cancer.** Scale bar: top row for 250 μm and bottom row for 100μm.

# Table S1. Primers used for RT-PCR

| Primer name Sequence（5’ to 3’） |
| --- |
| H-ATXN3-FO TGACGGGTCCAGAATTAATATCAG  H-ATXN3-RE TGCATCTGTTGGACCCTAATCA  H-Lgals9-FO ACCATTCTGGGAGGGCTGTA  H-Lgals9-RE GCGGACCACAGCATTCTCAT  M-ATXN3-FO GCCATAAGTCGCCAGGAAATC  M-ATXN3-RE CCTCCGCAGCTCTTCTGAAG  M-Lgals9-FO TTCAGTCGAGGCCAGAGCTT  M-Lgals9-RE CTGGATATCACCCGCCACTT  H-GAPDH-FO CATGTTCGTCATGGGTGTGAAA  H-GAPDH-RE GGCATGGACTGTGGTCATGAG  M-GAPDH-FO GCCACCCAGAAGACTGTGGAT  M-GAPDH-RE GGAAGGCCATGCCAGTGA |

# Table S2. Primers used for CRISPR

| Primer name Sequence（5’ to 3’） |
| --- |
| M1-ATXN3-FO CACCGCAATTGAGGATAGCTCCAC M1-ATXN3-RE AAACGTGGAGCTATCCTCAATTGC  M2-ATXN3-FO CACCGGCTCTAGCCATAAGTCGCC  M2-ATXN3-RE AAACGGCGACTTATGGCTAGAGCC  H1-ATXN3-FO CACCGATACTCTGGACTGTTGAAC  H1-ATXN3-RE AAACGTTCAACAGTCCAGAGTATC  H2-ATXN3-FO CACCGCTATTCAGCTAAGTATGCA  H2-ATXN3-RE AAACTGCATACTTAGCTGAATAGC |
